# Individualized selection of recent glucose monitoring devices for self-management based on competitive features

**DOI:** 10.12669/pjms.40.8.9855

**Published:** 2024-09

**Authors:** Olga Krylova, Sevara Marchenko, Alexandra Ermolaeva, Natalia Shustikova, Kristina Dyakonova

**Affiliations:** 1 Olga Krylova Associate Professor, Department of Pharmacy, I. M. Sechenov First Moscow State Medical University, Moscow, Russia; 2 Sevara Marchenko Associate Professor, Department of Organization and Management in the Field of Medicines Circulation, I. M. Sechenov First Moscow State Medical University, Moscow, Russia; 3 Alexandra Ermolaeva Associate Professor, Department of Clinical, Pharmacology and Propaedeutics of Internal Diseases, I. M. Sechenov First Moscow State Medical University, Moscow, Russia; 4 Natalia Shustikova, PhD of Medical Sciences Associate Professor, Deputy Dean for Educational and Organizational Work, Moscow University for Industry and Finance Synergy, Moscow, Russia; 5 Kristina Dyakonova Researcher, I. M. Sechenov First Moscow State Medical University, Moscow, Russia

**Keywords:** Blood glucose control, Blood glucose meter, Competitive features, Diabetes mellitus, Glucose monitoring devices, Self-management

## Abstract

**Objective:**

Goal of the study was to systematically review competitive advantages of medical devices for glucose monitoring in diabetic patients.

**Method.:**

The review is done systematically according to SALSA criteria and PRISMA guidelines. The search for eligible articles was held from February 16^th^ 2023 to March 1^st^ 2023 in Russian and English languages. The results were synthesized narratively, tabularly, and visually. The search was conducted in the following databases of scientific literature: PubMed, IEEE Xplore, Google Scholar, CyberLeninka, and eLibrary.

**Results.:**

Twenty-two out of fifty-two manuscripts met the inclusion criteria. The most promising and advantageous characteristics of the evaluated devices, as identified by researchers, include the following: the capability for noninvasive examination; features that facilitate use by patients with fine motor, hearing, and visual impairments; add-ons and software designed to improve patient compliance, including in pediatric populations; and device attributes that enhance the speed and accuracy of analysis while being free of iatrogenic effects.

**Conclusions.:**

With increasing prevalence of diabetes, glycemic control is crucial for preventing complications. The market offers numerous glucose monitoring devices (GMDs) with varying features, making selection challenging. Our study systematically categorized the strengths of each GMD model for diabetic patients, aiding informed device selection.

## INTRODUCTION

The incidence, prevalence and mortality rates of diabetes mellitus (DM) have been disappointingly rising for years worldwide.^1^,^2^ According to the 10^th^ edition of The International Diabetes Federation (IDF) Diabetes Atlas, 537 million adults between 20 and 79 years (every 10^th^) were diabetics in 2021 and this number is increase to selected to 643 million by 2030, 783 million by 2045^3^ and head over 1.3 billion by 2050 as the Global Burden of Disease states.^4^ The WHO determined DM as the 6^th^ leading cause of death among noncommunicable diseases in 2019 globally. Additionally, The IDF reports that 6.7 million deaths in 2021 were due to diabetes (every 5^th^ second) and at least 966 billion dollars were spent by the healthcare on the disease, what is more than tripling since 2006.^3^

Diabetes is one of the major causes of blindness, kidney failure, heart attacks and strokes, and lower limb amputations. DM and its associated complications place a serious economic burden on patients, their families, and public health systems.^1^,^2^ Under these circumstances the importance of glucose monitoring (GM) is reinforced. Considering how information technology allows remote doctor-patient interaction, it becomes extremely important for healthcare practitioners to facilitate self-monitoring of glucose for their patients, i.e. make it simple, accurate, and quick. This is where glucose-monitoring devices (GMD) come handy.

Clinical guidelines in various countries (China, Russia, India, UK, USA, Germany) mention two methods of glucose monitoring (GM) i.e., discrete and continuous (CGM).^5^-^10^ The former, traditional blood glucose monitoring (BGM) method involves the finger stick: a user pierces their finger, touches a drop of blood with a test strip on a blood glucose meter (glucometer), and receives the current glucose level in capillary blood. However, the latter method refers to a sensor implanted in the skin: a user attaches a CGM device (CGMD) to the upper arm or abdomen and retrieves glucose levels in subcutaneous tissue in real-time (real-time CGM, rtCGM) or at periodic intervals (intermittent scanning CGM, is CGM, also known as flash glucose monitoring, FGM).^11^ Real-time CGM devices (rtCGMDs) are also combined with insulin pumps into sensor-augmented pumps (SAP)^12^,^13^ and hybrid closed-loop systems (HCL), often referred to as “artificial pancreas” systems). Meanwhile, developers worldwide are striving to create reliable and commercially viable non-invasive glucose monitoring devices (GMDs) that utilize other body fluids,^14^ exhaled air and Raman spectroscopy,^15^ radio waves,^16^ and fluorescence.^17^

The development of science and technology inevitably leads to market launch of many different GMDs. Within the framework of personalized medicine, every professional physician should take into account the particularities of each of their patient with DM, and thus have an up-to-date and comprehensive understanding of features of the most researched and available GMDs.

The aim of the research was to conduct a systematic examination of the competitive benefits offered by medical devices designed for monitoring glucose levels in individuals with diabetes.

## METHODS

Choosing the review method, we relied on the underlying SALSA framework proposed by Grant M & Booth A in 2009^18^ and the PRISMA statement of 2020.^19^ From February 16, 2023 to March 1^st^, 2023 Dyakonova K, Yakimenko A, and Akhmetov B. conducted a comprehensive search via PubMed, IEEE Xplore in English, Google Scholar, CyberLeninka, eLibrary in Russian. Since the search in Google Scholar, CyberLeninka, and eLibrary was in Russian, we present keywords in Russian with Latin transliteration and English translation. The query for PubMed was aimed to retrieve full-text meta-analyzes, reviews, and systematic reviews sorted by best match that include keywords “blood glucose meter” and “diabetes” in the title and were published between 2018 and 2023. For IEEE Xplore the query was intended to receive public records containing keywords “diabetes”, “control” and “glucose meter” in all metadata of published articles from 2018 through 2023.

The query for Google Scholar was directed to obtain relevance-sorted open records containing keywords (an asterisk is used to embrace different word forms) “контрол* уровн* глюкозы” (kontrol* urovn* glyukozy, glucose level control), “медицинск* издели*” (medicinsk* izdeli*, medical devices) in any articles published from 2018 to 2023. In CyberLeninka we formed the query to retrieve full-text records that include keywords “glucose level control”, “diabetes”, and “medical devices” and were published between 2018 and 2023. Here we also utilized filter by OECD terms “клиническая медицина” (klinicheskaya medicina, clinical medicine), “медицинские технологии” (medicinskie tekhnologii, medical technology), “экономика и бизнес” (ekonomika i biznes, economics and business) and filter by scientific bases “BAK” (Higher Attestation Commission, HAC), “РИНЦ” (Russian Science Citation Index, RSCI), “Scopus”.

**Fig.1:**
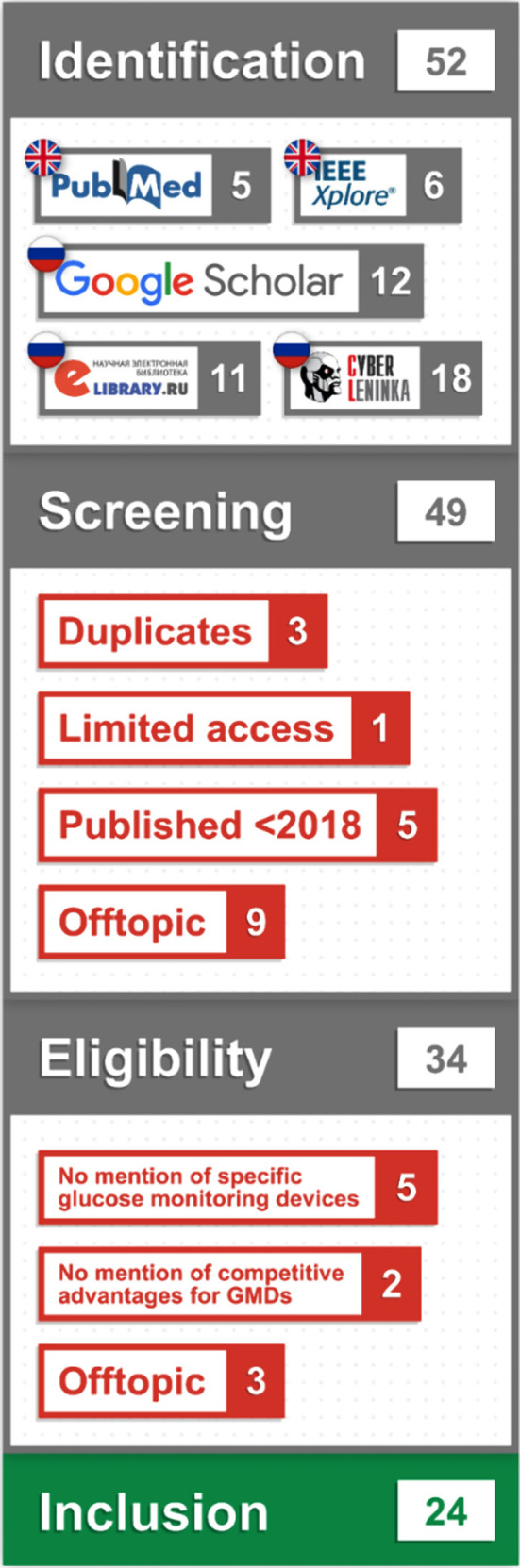
PRISMA flowchart.

For eLibrary the query was directed to select relevance-sorted (descending order) open full-text records containing the keywords “glucose” and “medical devices” in the title, abstract, and keyword section of journal articles and conference papers that were published between 2018 and 2023. Afterwards the investigation team selected articles, formed PRISMA flowchart (Fig.1), and checked the retrieved papers for the presence of sponsorship or employment at the manufacturer (key biases). To optimize the search and noting processes, the work was performed in a Google Sheet prepared in advance by Belosludtsev A. Together we synthesized the knowledge less narratively, mostly in tabular and graphical formats. The results of the review allow to understand what is known and what is available at the moment, as well as opportunities to optimize the review methodology and approaches to the selection of GMDs.

**Fig.2:**
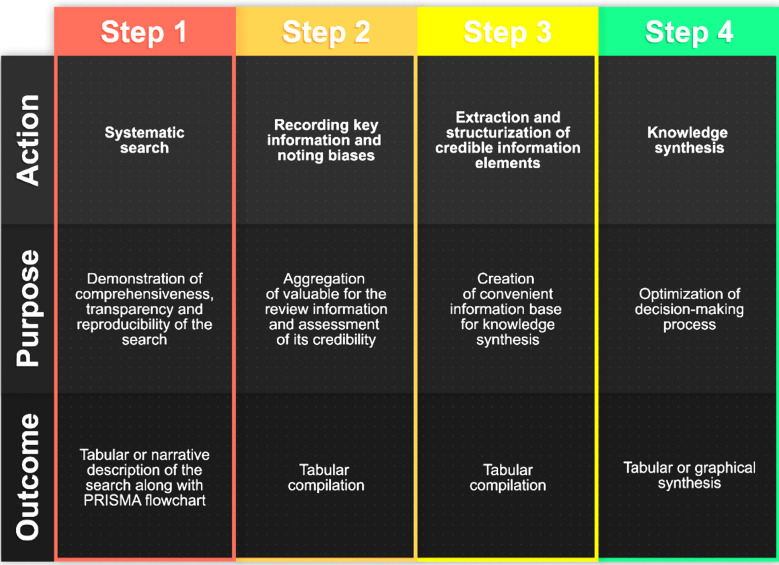
Action – Purpose – Outcome (APO) framework for the review.

In summary, the performed actions correspond to Grant & Booth’s “Systematized review” type. To foster mutual understanding we have come up with a complementary method to improve the demonstration of workflow and developed “Action – Purpose – Outcome” framework for the review (Fig.2). The framework may be applied in any work sequence that is shared publicly. We expect this way of presentation to accelerate knowledge translation.

## RESULTS

We have compiled key items from the included articles in Table-I. The table is a framework demonstrating the relationship between relevant information, its source’s details, and biases. A sponsorship or employment at the manufacturer are thought as main biases for the review as there is a chance of exaggerating positive feedback from authors. Information from such sources is taken as less credible. We suggest that presenting information in this way will facilitate more transparent research and faster analysis for readers. Thus, we hope the framework to find a strong place in future worldwide research practice.

**Table-I:** Key findings extracted from the included manuscripts.

2021	Detailed description of GMDs for different types of CGM, insulin pumps with built-in glucose measurement.^20^
2019	A description of the differences in accuracy between BGM and CGM. Advantages of CGM for the patient: reduced glycated hemoglobin, increased time in the target range.^21^
2020	Differences of glucose changes in blood and interstitial fluid are shown, which affects the accuracy of CGM and BGM.^22^
2019	CGM is not standard of care, nor is it generally recommended for inpatient use.^23^
2019	An innovative insulin management system consisting of an insulin pump and a personal diabetes manager is presented. The latter is a handheld device to wirelessly control the pump and may be paired with a BGMD.^24^
2020	The authors show a novel method for non-invasive BGM using diffuse reflection (remission) spectroscopy. However, to achieve a high level of sensitivity, it is necessary to improve the optical properties of semiconductor lasers, as well as to optimize the methods of processing the obtained data.^25^
2021	ARKRAY GLUCOCARD W features for outpatient and inpatient use are described. Features relevant to self-management are: no coding, micro sampling, compactness, swift analysis (5-8s), configurable sound notifications, memory capacity of 500 test results, USB data transfer to a computer, ISO 15197:2013 compliant accuracy, resistance to hematocrit fluctuations (20-70%), possibility to place explanatory marks (before or after meal) to describe reasons in glucose changes.^26^
2019	The three-color display of test results in glucose meters is described.^27^
2020	SelfyCheck Regular and SelfyCheck Prim have auto-coding for test strips and can also be used for data analysis.^28^
2018	The highlight of Contour Plus stands for a minimal deviation from the reference value of indicators.^29^
2021	The use of new LibreView technology (cloud-based web platform that automatically generates glucose level reports) with FreeStyle Libre FGM device (FGMD) significantly improves glycemic control, reduces the frequency of hypoglycemic episodes and is considered more cost-effective.^30^
2021	Advantages of application of the Contour Plus One glucometer are demonstrated: high accuracy of results, “second chance” technology and colored lighting.^31^
2022	New generation FGMDs make it possible to track data in a continuous mode, allowing to talk about chronic disease management.^32^
2019	Recent mHealth interventions targeting diabetes are diverse in their goals and components, and include insulin management applications, wearable GMDs, automated text messages, health diaries and virtual health coaching.^33^
2021	The advantages of Contour Plus One are the absence of coding, “second chance” technology, labels to mark the time of GM, informing about the compliance of glucose levels with the target range, the ability to store a large number of measurements.^34^
2020	The FreeStyle Libre provides better disease control as frequent GM minimizes the time a patient is hypo- or hyperglycemic and improves the average individual blood glucose level.^35^
2020	According to the cost-effectiveness analysis, the use of the Accu-Chek Performa glucometer in combination with the Accu-Chek Smart Pix (receiver, analyzer and transmitter of the data) is more efficient compared to the FreeStyle Libre instant GM technology.^36^
2018	The advantages of using a modern glucose meter Contour Plus in pediatric practice: compliance with the accuracy standard and “second chance” technology.^37^
2019	The IME-DC glucose meter is characterized by high accuracy and stability of measurements and can be used even in the smallest patients. Among the advantages of this medical device are promt results (within 10 seconds), a small amount of blood required (2 µl). The high quality sharpening of the needle makes it possible to make a single puncture to draw a sufficient amount of blood.^38^
2022	"No coding" (auto-calibration) technology (e.g., Contour TC, Contour Plus One, Diacont). Third generation non-invasive glucose meters do not require blood sampling and use of test strips (e.g., Omelon-1). Omelon A-1 also determines blood pressure and heart rate. Diacont Voice, Senso Card Plus, Senso Nova Plus have a voice control function. Contour Plus One has a color backlight.^39^
2021	Capillary BGM with the Farmaktiv CodeFree glucose meter correlated with venous BGM performed via Architect c8000 (r = 0.94; p < 0.001).^40^

## DISCUSSION

Analyzing the collected information, we identified five competitive feature groups based on their objectives. The first group focuses on accommodating individual peculiarities of human life, such as ophthalmic complications, impaired fine motor skills, diminished attention and memory, and child age, to increase the convenience of outpatient monitoring. The second group aims to simplify device handling to reduce iatrogenic errors, incorporating technologies like “no coding,” “sip-in,” and “second chance.” The third group prioritizes the accuracy of measurements by ensuring resistance to hematocrit and oxygen fluctuations and using multi-pulse technology. The fourth group centers on the efficient receipt, analysis, storage, and transmission of data. Finally, the fifth group seeks to make glucose monitoring accessible to everyone by making devices more cost-effective.

In order to save healthcare practitioners’ time, especially endocrinologists’, in the personal selection of GMDs, we deliver valuable knowledge through Table-II. The table plays as a basis for the infographics. The former item establishes contributions of the GMD’s features in diabetic’s life and health. The “Feature” column refers to the observed in the papers competitive advantages. “Aim” contains one of the aforementioned five feature groups. “Patient” describes diabetic’s life and health characteristics, i.e. for whom (target audience) this particular feature is thought to act as an advantage (problem-solver).

**Table-II:** Contributions of the GMD’s features in diabetic’s life and health.

Feature	Patient	If the feature is valid for GDM type		

		Glucometers	Continuous	Non-invasive
***Iatrogenic errors reduction***				
Auto-coding for test strips	all patients	+	-	-
***Convenience raise for a user***				
"Sip-in"	motor difficulties	+	-	-
"Second chance"	motor difficulties	+	-	-
Configurable sound notifications	visual impairments	+	-	-
Voice control	visual impairments	+	-	-
Safe strip disposal	motor difficulties	+	-	-
Micro-sampling	motor difficulties	+	-	-
Compactness	all patients	+	+	-
Swift analysis	all patients	+	+	-
Color display of test result	child age	+	-	-
***Data management optimization***				
USB data transfer to a computer	child age (parental benefits)	+	+	-
Automatic generation of glucose level reports	child age (parental benefits)	+	+	-
Labels to mark the time of GM	all patients	+	+	-
***Accuracy enhancement***				
Multi-pulse technology	all patients	+	-	-
Resistance to hematocrit fluctuations	all patients	+	-	-

## CONCLUSIONS

Nowadays when the incidence of diabetes is increasing, the need for glycemic control of patients remains one of the main factors in preventing complications of the disease. Due to the high demand for medical products for BGM, there are many models of GMDs from different manufacturers on the market. Each of them has both advantages and disadvantages and it is very difficult to make a choice in favor of a certain one. The result of our work is the systematization of the competitive advantages of each found model of GMDs in patients with DM. Our study can serve as a basis for making a decision about the use of a particular medical device.

This research offers valuable insights that can aid healthcare professionals and patients in making informed decisions regarding the adoption of a specific medical device for glycemic monitoring. Ultimately, our work aims to improve patient outcomes and enhance the management of diabetes through the selection of optimal GMDs tailored to individual needs and preferences.

### Authors’ Contributions:

**OK, SM & AE:** Investigation, Writing - Original Draft and Editing, Project administration.

**NS:** Methodology, Validation, Resources, Data curation, Writing - Review and Editing.

**KD:** Conceptualization, Supervision.
